# Plant hormones and phenolic acids response to UV-B stress in *Rhododendron chrysanthum* pall

**DOI:** 10.1186/s13062-024-00483-0

**Published:** 2024-05-28

**Authors:** Qi Sun, Xiangqun Li, Li Sun, Mingyi Sun, Hongwei Xu, Xiaofu Zhou

**Affiliations:** 1 Jilin Provincial Key Laboratory of Plant Resource Science and Green Production, Jilin Normal University, Siping, 136000 China; 2 Jilin Engineering Vocational College, Siping, China; 3 Siping Central People’s Hospital, Siping, China

**Keywords:** UV-B stress, Plant hormones, Phenolic acids, *Rhododendron chrysanthum* pall, Metabolome, Phosphorylated proteomics

## Abstract

Our study aims to identify the mechanisms involved in regulating the response of *Rhodoendron Chrysanthum* Pall. (*R.* *chrysanthum*) leaves to UV-B exposure; phosphorylated proteomics and metabolomics for phenolic acids and plant hormones were integrated in this study. The results showed that UV-B stress resulted in the accumulation of salicylic acid and the decrease of auxin, jasmonic acid, abscisic acid, cytokinin and gibberellin in *R.* *chrysanthum*. The phosphorylated proteins that changed in plant hormone signal transduction pathway and phenolic acid biosynthesis pathway were screened by comprehensive metabonomics and phosphorylated proteomics. In order to construct the regulatory network of *R.* *chrysanthum* leaves under UV-B stress, the relationship between plant hormones and phenolic acid compounds was analyzed. It provides a rationale for elucidating the molecular mechanisms of radiation tolerance in plants.

## Introduction

*Rhododendron chrysanthum* Pall. (*R. chrysanthum*), also known as cowhide tea, belongs to Rhododendron family and Rhododendron genus [1–3]. Perennial growth in low temperature and strong radiation, 1000–2506 m above sea level in alpine grassland or moss layer [2, 4, 5]. For a long time, UV-B is recognized as a potential stressor to organisms. In addition to morphological changes, it can also affect biological and genetic characteristics of plants [6, 7]. *R. chrysanthum* can maintain normal physiological activity and life process under high radiation conditions, indicating that it has a special physiological structure and mechanism of radiation tolerance, so *R. chrysanthum* has become a significant plant resource to study plant radiation tolerance [8–10].

Plant hormone, which is synthesized by the plant itself, is a small chemical that plays a key role in plant growth and development. Plant hormones interact with each other, which may not only cooperate with each other, but also antagonize each other. Hormones modulate plant growth and development and adapt for changing environmental conditions through complex interactions. Of the nine known phytohormones, abscisic acid (ABA), ethylene, salicylic acid (SA) and jasmonic acid (JA) are considered stress-responsive hormones; the others, including growth hormone, gibberellin (GA), cytokinin (CKS), oleuropein lactone (BRS) and solanum lactone (SLS) were categorized as growth-promoting hormones [11]. Numerous studies have demonstrated that each phytohormone does not have a single biological role in the plant, but plays a complex and effective role during different developmental stages, in different types of tissues or under various environmental conditions [12–15]. Combined with rich knowledge of plant hormones, this paper expounds the necessity of plant hormones in plant resistance to abiotic stress. For instance, exogenous treatment of ABA can alleviate damage under UV-B stress by maintaining normal development of *R. chrysanthum *[1]. Auxin (IAA) is recognized as a crucial modifier of plant life processes and also has a conservation effect on rice yield during high temperature stress [16].

Phenolic acids are a class of compounds with phenolic hydroxyl structure, which exist in almost all plant-derived foods. Phenolic acids are important for plant growth and development processes such as nutrient uptake, photosynthesis, protein synthesis, and cytoskeleton and structure formation [17]. Phenolic acids can also improve the stress resistance of plants and accumulate in plants under abiotic stresses such as low temperature, drought and ultraviolet radiation stress. Phenolic acid is a kind of secondary metabolites synthesized by shikimic acid pathway and phenylpropane metabolism [18]. Some studies have shown that continuous UV-B irradiation will affect plant photosynthesis, transpiration and pollination and cause cell damage. To avoid injury, plants synthesize flavonoids (such as anthocyanins) and phenolic acids to absorb UV-B and protect tissue [19].

Plant endogenous hormones can make use of their promoting effect on phenolic synthesis and improve plant antioxidation and other biological activities [20]. The results indicate that abscisic acid could regulate the root growth of maize seedlings under low temperature stress, and this regulation was achieved by increasing the activities of PAL and PPO, and then significantly increasing the contents of total phenols and total flavonoids in maize seedlings [21]. During the growth of radish seedlings, the expression of many genes was up-regulated after MeJA treatment, and then increased the synthesis of phenols [22]. Application of 0.1 and 0.2mM salicylic acid treatment at a certain concentration could increase the accumulation of phenolic compounds in wheat seedlings [23].

With the development of biotechnology, multi-omics techniques are more and more used to explain all kinds of biological phenomena, but there are still few studies on radiation tolerance of *R. chrysanthum*. In this experiment, hormone detection was combined with metabonomics and phosphorylated proteomics for the first time. At the level of phosphorylated protein and metabolism, differentially phosphorylated proteins, differentially accumulated metabolites and plant hormones in *R. chrysanthum* irradiated by UV-B were screened, and hormone signal transduction pathway and phenolic acid biosynthesis pathway were constructed. The purpose of this paper is to explore the regulation mechanism of plant hormones and phenolic acid compounds of *R. chrysanthum* under UV-B radiation, explain the possible role of *R. chrysanthum* in the process of adapting to the environment, and provide ideas for the study of plant resistance to UV-B radiation and the explanation of plant environmental adaptability.

## Results

### Changes in plant hormones in *R. chrysanthum* under UV-B stress

**Fig. 1:**
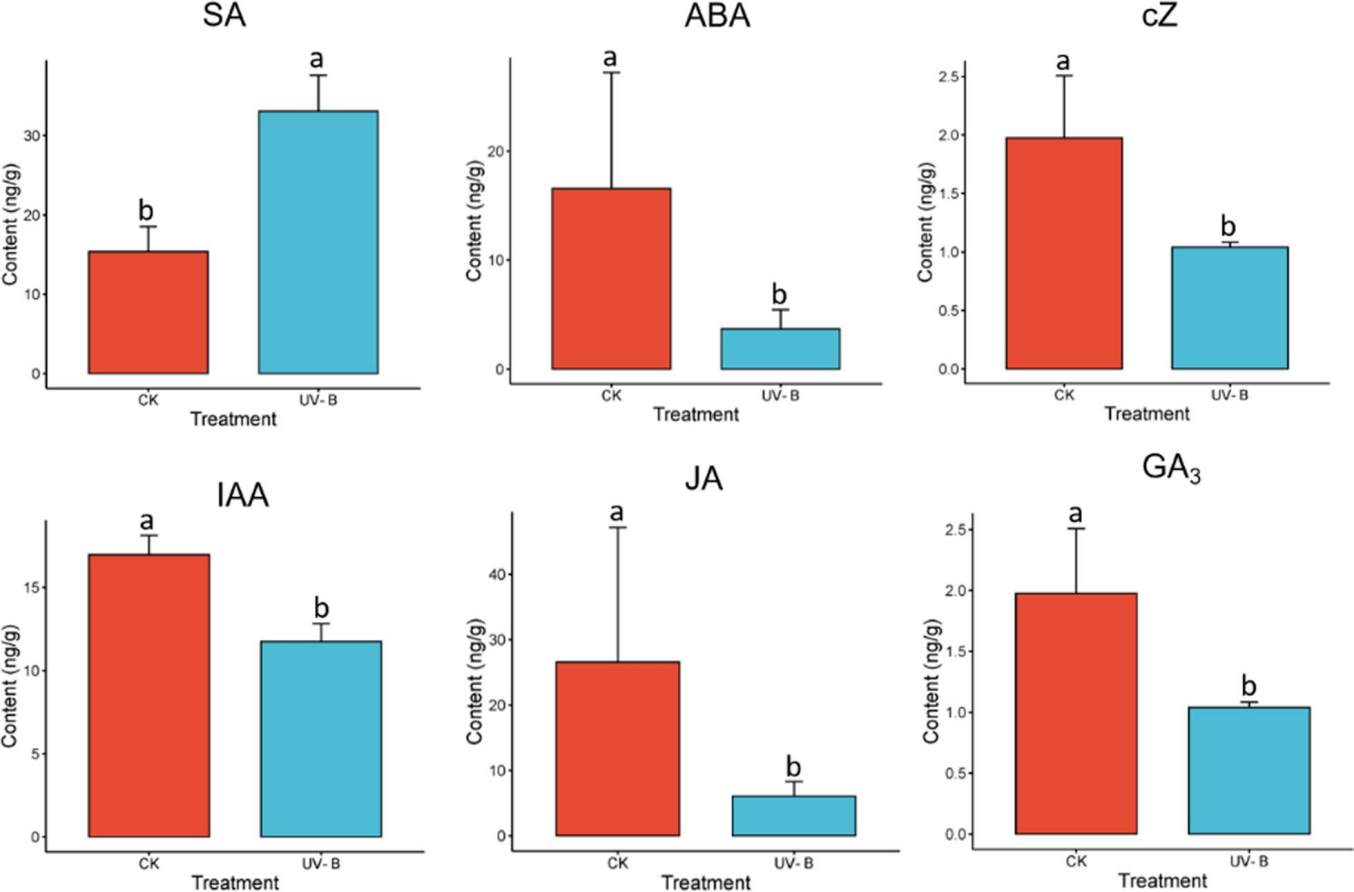
Plant hormone content validation results. CK: control group; UV-B: UV-B treated group. Auxin (IAA), salicylic acid (SA), cis-Zeatin (cZ), abscisic acid (ABA), Gibberellin A_3_ (GA_3_) and jasmonic acid (JA). Changes in phytohormone content. *Note* The data in the figure are the meanstandard error of three replicates. the letters indicate the significant difference between different treatments (*P* < 0, 05)

In the present study, ABA, JA, SA, IAA, ZA, cis-ZA (cZ) and GA_3_ were measured by LC—MS/MS to investigate the response mechanism of *R. chrysanthum* to UV-B stress (Fig. 1a). The results showed that the contents of IAA, ABA, Cz, JA and GA_3_ were found to be lower in the experimental group (UV-B) than in the control group (CK). SA contents in CK groups was lower than that in the UV-B group.

### Changes in phenolic acids in *R. chrysanthum* under UV-B stress

**Fig. 2:**
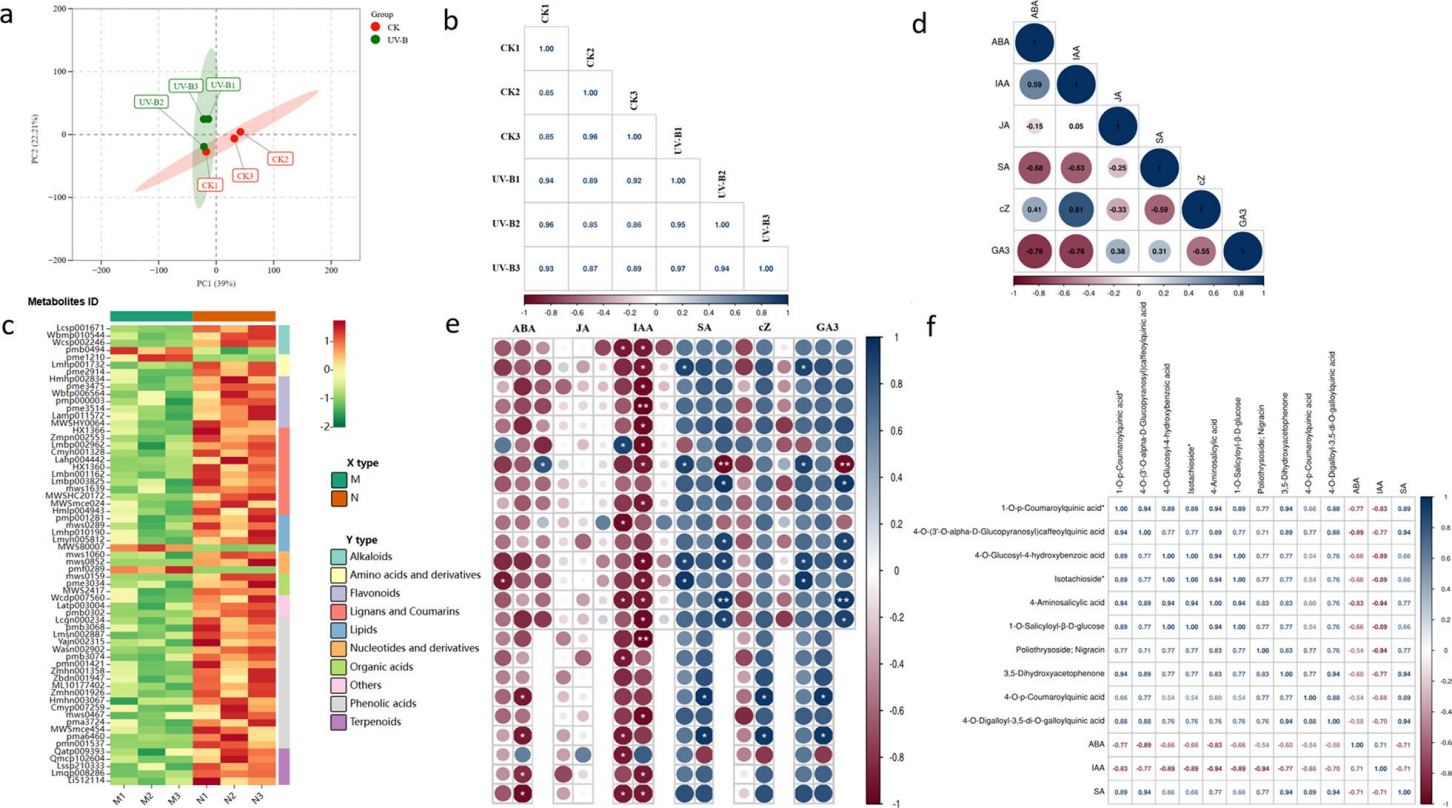
Global metabolome of *R. chrysanthum* in response to UV-B stress. **a** PCA analysis of *R. chrysanthum* under UV-B radiation; **b** differentially expressed metabolites (DEMs) correlation analysis; **c** Cluster analysis of differential metabolites of *R. chrysanthum* irradiated by UV-B. Six classes are evident upon clustering analysis. The colors of the boxes represent upregulated (red) and downregulated (green) metabolites; **d** correlation analysis between various plant hormones; **e** correlation analysis of plant hormones and metabolites; **f** correlation analysis of plant hormones and phenolic acids. *Note* Figures **b**, **d**, **e** and **f** were analyzed using the Spearman correlation coefficient calculation method, and the significant levels of *P*-Value were 0.05, 0.01 and 0.001 (i.e. *, ** and ***)

In order to determine the variation and relevance of metabolite accumulation patterns in *R. chrysanthum* under different treatments, the metabolite spectra of 6 samples were subjected to principal component analysis (PCA) (Fig. 2a). PCA demonstrated that the first principal component (PC1) accounted for 39.01% of the total variance, and the second principal component (PC2) accounted for 22.22%. The findings indicated that the metabolite accumulation pattern of *R. chrysanthum* under UV-B irradiation varied greatly. Furthermore, we performed a between-sample correlation analysis (Fig. 2b). The correlation coefficients between replicates of *R. chrysanthum* under different treatments were greater than 0.8. The results show that the metabolomics data obtained in this study are reliable.

To further analyze the distribution of metabolite abundance under UV-B stress, we performed hierarchical clustering of metabolites from treated and corresponding control plants (Fig. 2c). This analysis yielded five clusters that revealed different metabolic patterns in the stress treatments.

The interrelationships among the phytohormones were further elucidated by spearman correlation analysis, and the results are shown in Fig. 2d. ABA is negatively correlated with SA and GA_3_, IAA is negatively correlated with SA and GA_3_, and positively correlated with cZ. In this paper, Spearman correlation analysis of plant hormones and their metabolites was also carried out. The results are shown in Fig. 2e. The results showed that phenolic acids were highly correlated with plant hormones. Among them, 1-O-p-Coumaroylquinic acid*, 4-O-(3′-O-alpha-D-Glucopyranosyl) caffeoylquinic acid, 4-O-Digalloyl-3,5-di-O-galloylquinic acid, 3,5-Dihydroxyacetophenone, 4-O-p-Coumaroylquinic acid, 4-Aminosalicylic acid, 4-O-Glucosyl-4-hydroxybenzoic acid, Isotachioside, 1-O-Salicyloyl-β-d-glucose and Poliothrysoside; Nigracin were highly correlated with hormone ABA, IAA and SA (Fig. 2f, Table 1).

**Table 1:** Statistical analysis of the relationship between *R. chrysanthum* phenolic acid compounds and plant hormones under UV-B radiation

Phytohormone	Compounds	Formula	Correlation coefficient values	Structure
SA	1-O-p-Coumaroylquinic acid	C_16_H_18_O_8_	0.89**	
	4-O-(3'-O-alpha-D-Glucopyranosyl) caffeoylquinic acid	C_22_H_28_O_14_	0.94**	–
	4-O-Digalloyl-3,5-di-O-galloylquinic acid	C_35_H_28_O_22_	0.94**	
	3,5-Dihydroxyacetophenone	C_8_H_8_O_3_	0.94**	
	4-O-p-Coumaroylquinic acid	C_16_H_18_O_8_	0.89**	
ABA	4-O-(3'-O-alpha-D-Glucopyranosyl) caffeoylquinic acid	C_22_H_28_O_14_	− 0.89**	–
	4-Aminosalicylic acid	C_7_H_7_NO_3_	− 0.83**	
IAA	4-O-Glucosyl-4-hydroxybenzoic acid	C_13_H_16_O_8_	− 0.89**	
	Isotachioside	C_13_H_18_O_8_	− 0.89**	–
	4-Aminosalicylic acid	C_7_H_7_NO_3_	− 0.94**	
	1-O-Salicyloyl-β-D-glucose	C_13_H_16_O_8_	− 0.89**	–
	Poliothrysoside; Nigracin	C_20_H_22_O_9_	− 0.94**	–

Note: Spearman correlation coefficient was used to calculate the correlation between hormones and phenolic acids, and the significant levels of *P*-Value were 0.05, 0.01 and 0.001(i.e. *, ** and ***), respectively

### Phosphorylated proteomics analysis of *R. chrysanthum* under UV-B stress

**Fig. 3:**
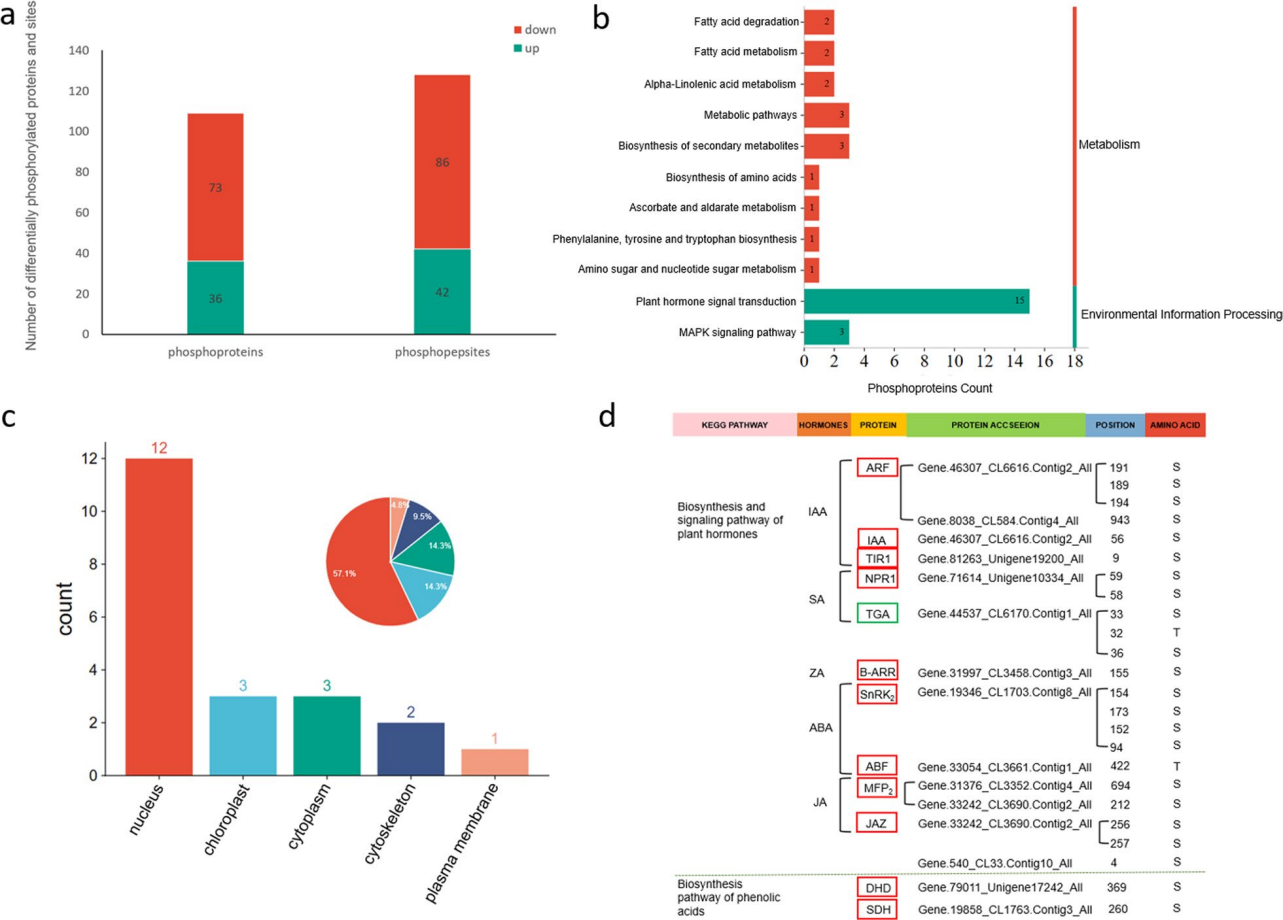
Phosphorylation proteome analysis of *R. chrysanthum* under UV-B irradiation. **a** Quantitative analysis of differentially phosphorylated proteins and loci; **b** KEGG classification of phosphorylated proteins in the plant hormone signal transduction pathway and biosynthesis pathway of phenolic acids; **c** Subcellular localization of phosphorylated proteins in plant hormone signal transduction pathways and biosynthesis pathway of phenolic acids; **d** Phosphorylation sites of plant hormone signal transduction pathway and biosynthesis pathway of phenolic acids

To delve into the potential regulatory networks of hormones and metabolism under UV-B stress in *R. chrysanthum*. TMT labeling and phosphorylation modification enrichment techniques as well as high-resolution liquid chromatography-mass spectrometry quantitative proteomics research strategies were used to quantify the protein phosphorylation of *R. chrysanthum* under UV-B stress. We identified 2,872 phosphopeptides from 2,508 phosphoproteins, respectively. Of these, a total of 128 phosphorylation sites were identified on 109 differentially phosphorylated proteins (*p* < 0.05, fold change > 1.2) (Fig. 3a). According to KEGG enrichment analysis, 15 proteins were enriched in plant hormone signal transduction, and 3 proteins were enriched in MAPK signaling pathway, metabolic pathways and biosynthesis of secondary metabolites, respectively (Fig. 3b). After UV-B stress, phosphorylation levels increased at 42 sites and decreased at 86 sites. The subcellular localization of differentially expressed phosphorylated proteins in the *R. chrysanthum* phytohormone signaling and phenolic acid biosynthesis pathways (Fig. 3c). We found that 57.1% of the differentially phosphorylated proteins were concentrated in the nucleus and 14.3% in the cytoplasm, with the highest number of differentially phosphorylated proteins located in the nucleus.

In Fig. 3d, the phosphorylated proteins and changed sites in hormone signal transduction pathway after UV-B radiation were screened. A total of twenty-two phosphate sites were screened. Among the IAA signal transduction pathways, four sites were phosphorylated in ARF (S191, S189, S194, S943), one in IAA (S56), one in TIR1 (S9), two in NPR1 (S59, S58), three in TGA (S3, T32, S36), and one in B-ARR (S155) in ZA signal transduction pathway. In the ABA signal transduction pathway, four sites were phosphorylated in SNRK2 (S154, S173, S152, S94), one in ABF (T422), two in MFP2 (S694, S212), and three in JAZ (S256, S257, S4) in JA signal transduction pathway. In the biosynthesis pathway of phenolic acids, one site were phosphorylated in DHD (S369), one in SDH (T260).

### Expressions of relevant DEPs in plant hormone signal transduction and phenolic acid biosynthesis pathway

**Fig. 4:**
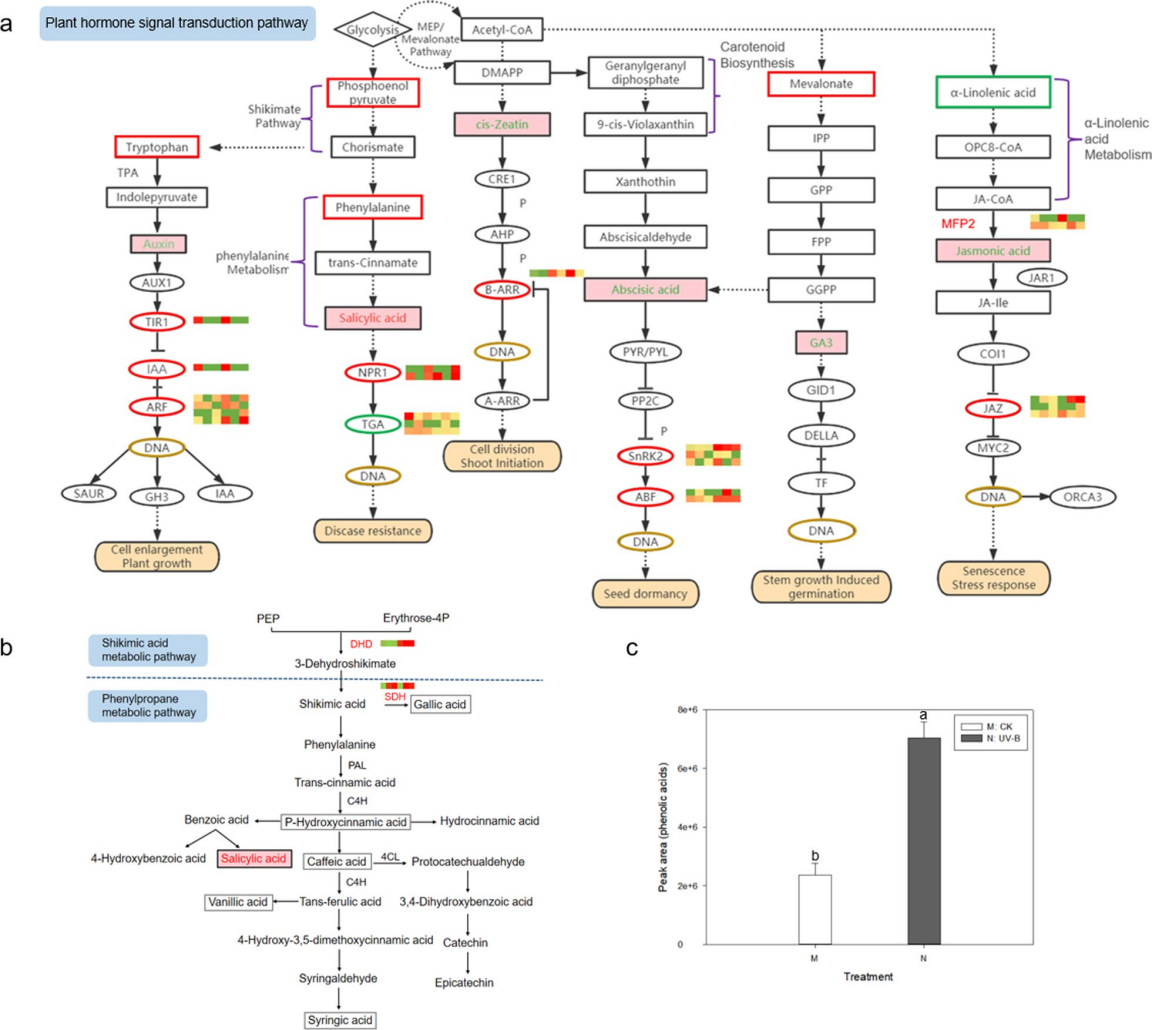
Metabolic pathway analysis of *R. chrysanthum* under UV-B stress. **a** Biosynthesis and signal transduction pathways of plant hormones (From left to right are auxin, salicylic acid, zeatin, abscisic acid, GA3 and jasmonic acid. Boxes represent metabolites, ellipses represent phosphorylated proteins, and thermograms show changes in protein content, with red indicating up-regulation and green indicating down-regulation. **b** Biosynthesis pathway of phenolic acids (The heat map shows the change of phosphorylated protein,and the gray box indicates that phenolic acids have been detected but have no significant change.); **c** Change in total phenolic acid content. *Note* The data in the **c** are the meanstandard error of three replicates. the letters indicate the significant difference between different treatments (*P* < 0, 05)

Based on the phytohormone data we have discussed from above, the metabolic and signaling pathways are shown in Fig. 4a. As results of ABA showed, two serine/threonine-protein kinase (SnRK2), and four ABA responsive element binding factor (ABF) proteins phosphorylation levels were increased after UV-B treatment compared to the untreated group. Distinctly, SnRK2 and ABF proteins were up-regulated under UV-B stress. For SA, the expressions of the protein phosphorylation level of regulatory protein NPR1was proved with enhancements under UV-B stress. However, transcription factor TGA protein decreased. In the JA relative pathway, UV-B stress increased the expression values of the MFP2 and JAZ proteins phosphorylation levels when compared to CK. Furthermore, under UV-B stress, we verified significant expression differences in the phosphorylation levels of B-ARR proteins in cZ biosynthesis and signaling pathways. In the IAA-related pathway, the phosphorylation levels of three related proteins were confirmed to be up-regulated in the UV-B-treated group compared with the untreated group. For GA3, there are no changes in protein phosphorylation levels in biosynthesis and signal transduction pathway.

In view of the fact that phenolic acids play an important role in plant response to abiotic stress, we comprehensively analyzed the changes of protein phosphorylation in phenolic acid biosynthesis pathway in chrysanthemum leaves under UV-B stress. We found that DHD and SDH were mostly up-regulated (Fig. 4b). Abiotic stress can lead to the increase of phenolic acid level in plants, thus improving plant cold tolerance. For this reason, we measured the changes of phenolic acid content in plants before and after UV-B stress. From the experimental results we were able to see that UV-B radiation significantly increased the phenolic acid content of the plant (Fig. 4c).

## Discussion

Plant hormones play a central role in the whole life cycle of plants, and play a synergistic or antagonistic role under abiotic stress. They can quickly perceive changes in the environment[24].

### Response of auxin in *R. chrysanthum* to UV-B stress

Auxin, as a kind of growth-promoting hormone, participates in the perception and defense of salt stress signals in plants, thus improving the tolerance of plants to abiotic stress [25]. Phosphorylated proteome results showed that these three proteins differed significantly among treatments and were all up-regulated in expression under UV-B stress. Transport inhibitor response protein 1(TIR1) is an important protein for plants to respond to auxin signals [26]. After auxin binds to the receptor TIR1 protein, the F-box protein encoded by TIR1 can form a SCFSKPI/cullin/F-box-type 26S ubiquitin protease complex with S-phase kinase-related protein 1(SKPI) analogues ASK1 and Cullin, which interacts with auxin and Aux/IAA transcription factors. It promotes the degradation of Aux/IAA protein after ubiquitination modification, and then activates the genes related to auxin response [27, 28]. Previous studies have shown that overexpression of MiR393-TIRI gene in Arabidopsisthaliana may trigger auxin-mediated downstream pathway, and enhance the salt stress resistance of plants through osmotic regulation and increasing Na excretion, thus significantly enhancing the salt resistance of transgenic Arabidopsis thaliana [29]. IAA proteins are a group of transient transcriptional regulatory proteins that interact with ARFs. Auxin response factor ARF is a unique family of auxin response transcription factors in plants, which not only plays an important role in auxin signaling pathway, but also has an important influence on signal transduction of other plant hormones [30]. Most ARF of *R. chrysanthum* was positively regulated under UV-B, which indicated that the consumption of auxin increased under UV-B radiation.

### Response of salicylic acid in *R. chrysanthum* to UV-B stress

In addition to playing a key part in plant defense against pathogens, SA may protect plants to abiotic stresses by different stress signaling pathways [31]. When plants are stimulated by the outside world, they can induce plants to affect the SA expression level through self-regulation of hormone signal transduction and biosynthesis [32]. Sharma et al. showed that ozone induced the production of salicylic acid in Arabidopsis leaves, which was also induced by H2O2, proving that the activation of self-defense response was closely related to the salicylic acid-dependent pathway [33]. Throughout the current results of phosphorylated proteome, the expression of NPR1 was up-regulated and TGA was down-regulated after UV-B radiation. The existence of cold-induced NPRI accumulation and cold domestication in Arabidopsis was also previously demonstrated [34]. However, NPRl is not the only receptor for SA signaling, which suggests a low correlation between its concentration and protein expression due to the characterization of SA in phytohormones. The transcription factor TGA belongs to the bZIP family and was consistently down-regulated in the stress group compared to the CK group.

### Response of cytokinin in *R. chrysanthum* to UV-B stress

Cis-zeatin (cZ), a cytokinin compound, is a plant regulator in dealing with abiotic stresses such as drought, salinity and heat [35].

Cytokinin can also be used as a signal substance, and cytokinin signal transduction is carried out by membrane associated proteins [36]. All cytokinin receptors share a common domain outside the predicted cytoplasm, which is the putative binding site of the cytokinin molecule [37]. When stimulated, the HK receptor self-phosphorylates and transfers the phosphate group to AHPs, which contains histidine phosphate transporter, so that AHPs is transported from cytoplasm to the nucleus and to B-RRs. In the nucleus, the phosphorylation of class B receptors can not only promote the transcription of TypeA-Rs, A regulatory factor of type A response, but also promote the transcription of various proteins. Type A RRs is an important endogenous suppressor, which negatively regulates cytokinin signaling [38]. Rashotte et al. showed that ARR22, as a member of the AP2-erebp transcription factor family, has a strong CK signaling function and its expression is up-regulated after CK processing. These genes encode so-called CK response factors (CRFs), which accumulate rapidly in the nucleus and whose downstream targets overlap with type B ARRs, suggesting that both CRFs and type B ARRs mediate CK responses [39].

ARR-B is a positive regulator of cytokinin signal, and the expression of ARR-B is only up-regulated under UV-B stress [40]. Therefore, the expression of ARR-B in UV-B group increased the positive regulation of cytokinin signal, thus decreased auxin to maintain the radiation tolerance of *R. chrysanthum* seedlings.

### Response of gibberellin in *R. chrysanthum* to UV-B stress

GA_3_ can increase the extensibility of cell wall and promote the germination of dormant buds. In the process of gibberellin synthesis, GGDP synthesized GA12 through multi-step reaction, and GA20ox and GA3ox catalyzed the formation of different active forms of gibberellin [41]. GA interacts with GID1, and the GA-GID1 complex interacts with the gibberellin inhibitory protein DELLA and recruits the E3 ubiquitin ligase SCFGID2/SLY1 to degrade DELLA protein specifically by the 26S proteasome, thereby eliminating the inhibition of gibberellin signaling pathway [42]. Many studies have shown that gibberellin, like other resistance hormones, can regulate plant growth and development and resist external environmental stress [43]. Without phosphorylated proteins were found in this experiment, but other reports have shown that gibberellin can regulate plant growth and development and stress together with other hormones.

### Response of abscisic acid in *R. chrysanthum* to UV-B stress

ABA has always been considered as a hormone conducive to plant tolerance to abiotic stress. Stress may affect ABA biosynthesis and signal transduction processes, and the right amount of ABA application is crucial to plant tolerance.

At present, the core components of ABA signal transduction have been identified by molecular genetics and biochemical methods, and have been further confirmed by a large amount of genetic and structural evidence [44]. Under stress conditions, existing PP2Cs can interact with SnRKs and dephosphorylation deactivates them [45]. The influence of external environment will cause the increase of abscisic acid content in plants, and at the same time, it is recognized by receptor proteins PYR/PYL/RCARs, and form receptor complexes with them to prevent the dephosphorylation of SnRKs and activate SnRKs [46]. The latter action activates downstream members in response to the expression of ABA signaling target genes and related regulatory genes. Kagaya et al. have shown that rice homologous protein TRAB1 can be phosphorylated by SAPK and rapidly phosphorylated in response to ABA to resist the damage to plants caused by external stress [47]. Furihata et al. have demonstrated that ABA in plants can activate SnRK2 protein kinase and regulate ABA signaling through phosphorylation and dephosphorylation of multiple sites in AREB conserved regions [48].

However, our findings suggest that ABA content decreased after UV-B radiation, which may be related to the adaptive tolerance of *R. chrysanthum* grown in alpine environments. Furthermore, in this study, most of the SnRK2s proteins were enhanced under stress, which is consistent with the study that ABA can activate SnRK2s [49]. Furthermore, in addition to the presence of phosphorylation of ABF by SnRK2 protein kinase, the report article suggests another direct regulation of ABF by ABA [49]. The results showed that the ABFs were enhanced after temperature treatment. These findings suggest that accumulated ABA has a central function in controlling the outgrowth of downstream signaling genes.

### Response of jasmonic in *R. chrysanthum* to UV-B stress

A major role is played by JA in the development of plants from germination to senescence [50]. It also activates the defense mechanism of plants against environmental stress.

When the plant is in normal environment, JAZs can interact with COI1 protein and form co-receptors sensing JA signal to recognize JAIle and inhibit MYC2 transcription factor activity [51]. When plants are subjected to abiotic stress, endogenous JA will accumulate, release the inhibition of transcription factor MYC2, activate downstream target genes, and enhance plant stress resistance [52]. JA also interacts with other signaling molecules to trigger JA signals in plants and promote downstream gene expression through JA signal transduction pathways. Studies have found that under stress conditions, the level of JA increases, JAR1 induces JA-ILE to produce JA-ILE, binds JAZ to SCFCOI complex, releases the transcriptional inhibition of MYC2 by JAZ, activates downstream genes, regulates various metabolic pathways, and synthesizes a series of secondary metabolites. JA is the main substance used by plants to resist natural enemies. Its signal transduction pathway is an important pathway regulating the synthesis of plant secondary metabolites, which can organically link the effects of different environmental factors with a series of physiological indicators, and play an important role in coping with adversity [53].

The results of phosphorylated proteome showed that the expression of JAZ and MFP2 in the treatment group was up-regulated, which was theoretically beneficial to the accumulation of JA. However, the up-regulation of JAZ may have accelerated the JA depletion; therefore, the content decreased significantly.

In addition, Spear-man correlation analysis was performed to elucidate the relationships among plant hormones, and the results are shown in Fig. 2d. The results showed that growth hormone was significantly and positively correlated with cytokinin with a correlation coefficient of 0.81. These new findings are helpful to clarify the plant hormone network under temperature stress.

### Response of phenolic acid in *R. chrysanthum* to UV-B stress

Plant phenolic acid is a kind of compounds with phenolic hydroxyl structure, and its precursors are generally derived from the intermediates of glycolysis and pentose phosphate pathway [54]. shikimic acid was synthesized from phosphoenolpyruvate and erythritose-4-phosphate as substrates through shikimic acid pathway, and then polyphenols were synthesized through phenylpropane metabolism pathway, which play an essential part in plant growth and development and adaptation to abiotic stress [55]. The results showed that UV-B stress significantly increased the content of total phenolic acids and the phosphorylation level of DHD and SDH proteins. Plant endogenous hormones can make use of their promoting effect on phenolic synthesis and improve plant tolerance and other biological activities. In phenolic acid biosynthesis pathway, hydrochloric acid increases after UV-B radiation. Salicylic acid is a kind of exogenous signal molecule, which can promote plant growth, regulate seed germination, regulate ethylene synthesis, induce plant defense responses such as disease resistance, drought resistance and salt resistance, and regulate various synthetic metabolic processes of plants through a variety of ways, so as to improve metabolites.

In this work, Spearman correlation analysis of plant hormones and phenolic acids was also carried out. The results are shown in Fig. 2f. The correlation coefficients between 1-O-p-Coumaroylquinic acid*, 4muro-(3′-O-alpha-D-Glucopyranosyl) caffeoylquinic acid, 4murine Omuryl and 4-O-p-Coumaroylquinic acid and SA were 0.89, 0.94, 0.94 respectively, showing a positive correlation. 4-O-p-Coumaroylquinic acid exists in plants. It is one of the important hormones to promote plant growth and development. It can promote the flow of protoplasts, accelerate the speed of plant roots, elongate stems and expand leaves [56]. The changes of ABA and IAA were negatively correlated with several phenolic acids. The results provide a new idea for revealing the interaction between phenolic acids and plant hormones, and contribute to the construction of the regulatory mechanism of *R. chrysanthum* seedlings under UV-B stress.

## Materials and methods

### Plant materials and treatment

The *R. chrysanthum* was preserved in the artificial climate room and cultured in the photon flux 50umoL/(m^2^ s) of 18 °C and 16 °C (day/night). The tissue culture seedlings of cowhide du Peng, which had the same growth state for 8 months, were selected as the research material [19]. The experimental materials were divided into experimental group (UV-B) and control group (CK), and the experimental group was irradiated with 8 h/d under UV-B for two days, a total of 12 groups of samples. In the control group, the co-sampling strategy was used to eliminate the differences among individuals after PAR irradiation for 48 h, and three biological repeats were carried out in this study. The treatment of PAR irradiation was to put 400 nm light film (Edmund; Filter Long2IN SQ, USA) on the culture flask. UV-B radiation treatment is to measure the filter (Edmund; Filter Long2IN SQ, USA) of 295 nm on the culture flask. The artificial radiation UV-B (Philips, Ultraviolet-B TL 20W/01 RS, Amsterdam, NY, The Netherlands). The effective receiving irradiance of the sample is UV-B: 2.3 W/m^2^, PAR: 50 umol/ (m^2^.s) [8, 57].

### Plant hormone detection

#### Liquid chromatography-tandem mass spectrometry detection

The LC–MS/MS detection in this study is based on MetwareDatabase, a self-built database of Wuhan Metville Company. HPLC grade acetonitrile (ACN) and methanol (MeOH) were purchased from Merck (Darmstadt, Germany). MilliQ water (Millipore, Bradford, USA) was used in all experiments. All of the standards were purchased from Olchemim Ltd. (Olomouc, Czech Republic) and isoReag (Shanghai, China). Acetic acid and formic acid were bought from Sigma-Aldrich (St Louis, MO, USA). The stock solutions of standards were prepared at the concentration of 1 mg/mL in MeOH. All stock solutions were stored at − 20 °C. The stock solutions were diluted with MeOH to working solutions before analysis. After the mass spectrum data of each sample is obtained, the chromatographic peaks of the substance to be measured are combined and quantitative determination is performed by internal standard method.

#### Sample preparation

Take 50 mg of the ground sample, add the internal standard mixed solution and extraction agent, and stir for 10 min to make it fully mixed. Centrifuge at 4 °C, 12000r/min for 5 min, concentrate the supernatant, then re-dissolve it with methanol/aqueous solution, filter by filter membrane, and put the sample into the sample bottle for LC–MS/MS detection [58].

#### Qualitative and quantitative of plant hormones

MetwareDatabase, which is based on standard products, qualitatively analyzes the data of mass spectrum detection. Quantitative analysis was carried out by multi-reaction monitoring mode analysis with triple four-bar mass spectrometry.

#### Standard curve and absolute quantification

The standard solution with different concentrations of 0.01 ng/mL, 0.05 ng/mL, 0.1 ng/mL, 0.5 ng/mL, 1 ng/mL, 5 ng/mL, 10 ng/mL, 50 ng/mL, 100 ng/mL, 200 ng/mL and 500 ng/mL were prepared respectively (TRP and SAG were above) 20 times of the concentration, that is, the standard curve concentration range is 0.2–10000 ng/mL), obtain the chromatographic peak intensity data of the corresponding quantitative signal of each concentration standard product, and draw the standard curve, and the content of hormones in the sample (ng/g).

### Identification and quantification of metabolites

#### Dry sample extraction

Using vacuum freeze-drying technology, place the biological samples in a lyophilizer (Scientz-100F, ningbo), then grinding (30 Hz, 1.5 min) the samples to powder form by using a grinder (MM 400, Retsch, Germany). Next, weigh 50 mg of sample powder using an electronic balance (MS105DΜ) and add 1200 μL of − 20 °C pre-cooled 70% methanolic aqueous internal standard extract (less than 50 mg added at the rate of 1200 μL extractant per 50 mg sample). Vortex once every 30 min for 30 s, for a total of 6 times. After centrifugation (rotation speed 12,000 rpm, 3 min), the supernatant was aspirated, and the sample was filtered through a microporous membrane (0.22 μm pore size) and stored in the injection vial for UPLC-MS/MS analysis.

#### UPLC conditions

The sample extracts were analyzed using an UPLC-ESI–MS/MS system (UPLC, ExionLC™ AD, https://sciex.com.cn/) and Tandem mass spectrometry system (https://sciex.com.cn/). The analytical conditions were as follows, UPLC: column, Agilent SB-C18 (1.8 µm, 2.1 mm × 100 mm); The mobile phase was consisted of solvent A, pure water with 0.1% formic acid, and solvent B, acetonitrile with 0.1% formic acid. Sample measurements were performed with a gradient program that employed the starting conditions of 95% A, 5% B. Within 9 min, a linear gradient to 5% A, 95% B was programmed, and a composition of 5% A, 95% B was kept for 1 min. Subsequently, a composition of 95% A, 5.0% B was adjusted within 1.1 min and kept for 2.9 min. The flow velocity was set as 0.35 mL per minute; The column oven was set to 40 °C; The injection volume was 2 μL.

Mass spectrometry conditions mainly include:

Electrosprayionization source (ESI) temperature is 500 °C; Ion spray voltage (IS)5500 V (positive ion mode)/− 4500 V (negative ion mode); The ion source gas I(GSI), gas II(GSII) and gas curtain gas (CUR) were set to 50, 60 and 25psi respectively, and the collision-induced ionization parameters were set to high. QQQ scanning uses MRM mode, and the collision gas (nitrogen) is set to medium. Through further optimization of declusteringpotential (DP) and collisionenergy (CE), the DP and CE of each MRM ion pair are completed. According to the metabolites eluted in each period, a specific set of MRM ion pairs was monitored in each period. The effluent was alternatively connected to an ESI-triple quadrupole-linear ion trap (QTRAP)-MS.

#### ESI-Q TRAP-MS/MS

The ESI source operation parameters were as follows: source temperature 500 °C; ion spray voltage (IS) 5500 V (positive ion mode)/-4500 V (negative ion mode); ion source gas I (GSI), gas II(GSII), curtain gas (CUR) were set at 50, 60, and 25 psi, respectively; the collision-activated dissociation (CAD) was high. QQQ scans were acquired as MRM experiments with collision gas (nitrogen) set to medium. DP (declustering potential) and CE(collision energy) for individual MRM transitions was done with further DP and CE optimization. A specific set of MRM transitions were monitored for each period according to the metabolites eluted within this period [59].

### Phosphorylated proteomic analysis

#### Protein extraction and trypsin digestion

Appropriate tissue samples were weighed and thoroughly ground into powder. In each group, four times the amount of powder phenol ex-traction buffer was added, and the samples were ultrasonically split. Equal volume of Tris was added to equilibrate the phenol and centrifuged at 5500 Rpm for 10 min at 4 °C. A 5 × concentration of 0.1 M ammonium acetate/methanol was added to the supernatant overnight, and the precipitate was washed with methanol and acetone. Finally, the precipitation was re-dissolved with 8 M urea and the protein concentrations were determined using a BCA kit.

To the protein solution, dithiothreitol was added and reduced for 30 min at 56 °C to a final concentration of 5 mm. After that, the final concentration of iodoacetamide was 11 mM, and 15 min was incubated at room temperature without light. Finally, the sample was diluted to a concentration of urea of less than 2 m. Trypsin was added and hydrolyzed overnight at 37 °C at a mass ratio of 1:50 (trypsin: protein). Then trypsin (trypsin: protein) at a mass ratio of 1:100 was added and enzymatic digestion was continued for 4 h.

#### TMT/iTRAQ labeling and LC–MS/MS analysis

StrataXC18 (Phenomenex) was used to desalinate enzymic peptides and freeze-dry them under vacuum. 0.5MTEAB was used to dissolve the peptide and label the peptide as instructed by the TMT kit manufacturer.

The tryptic peptides were dissolved in 0.1% formic acid (solvent A), directly loaded onto a home-made reversed-phase analytical column (15-cm length, 75 μm i.d.). The gradient was comprised of an increase from 6 to 23% solvent B (0.1% formic acid in 98% acetonitrile) over 26 min, 23–35% in 8 min and climbing to 80% in 3 min then holding at 80% for the last 3 min, all at a constant flow rate of 400 nL/min on an EASY-nLC 1000 UPLC system. The peptides were subjected to NSI source followed by tandem mass spectrometry (MS/MS) in Q ExactiveTM Plus (Thermo) coupled online to the UPLC [1].

### Determination of total phenols in *R. chrysanthum*

The 0.5 g *R. chrysanthum* leaves were crushed in the mixture of 1% HCl-methanol, and the homogenate was collected and fixed in the gradient test tube of 20 ml. Rinse the mortar with 1% HCI-methanol solution, transfer it to the test tube, and extract 20 min from light at 4℃. The 1% HCI-methanol solution was used as a blank reference to zero, and the filtrate was taken to determine the absorbance value of the solution at the wavelength 280 nm and repeated 3 times (spcetrum, shanghai).

### Statistical analysis

The heatmap, correlation coefficient matrix diagram and bar chart were plotted via online website: http://www.bioinformatics.com.cn with modifification. PCA diagram was analyzed and drawn using https://www.metaboanalyst.ca/. Statistical analyses was conducted using IBM SPSS statistical software.

## Conclusions

**Fig. 5:**
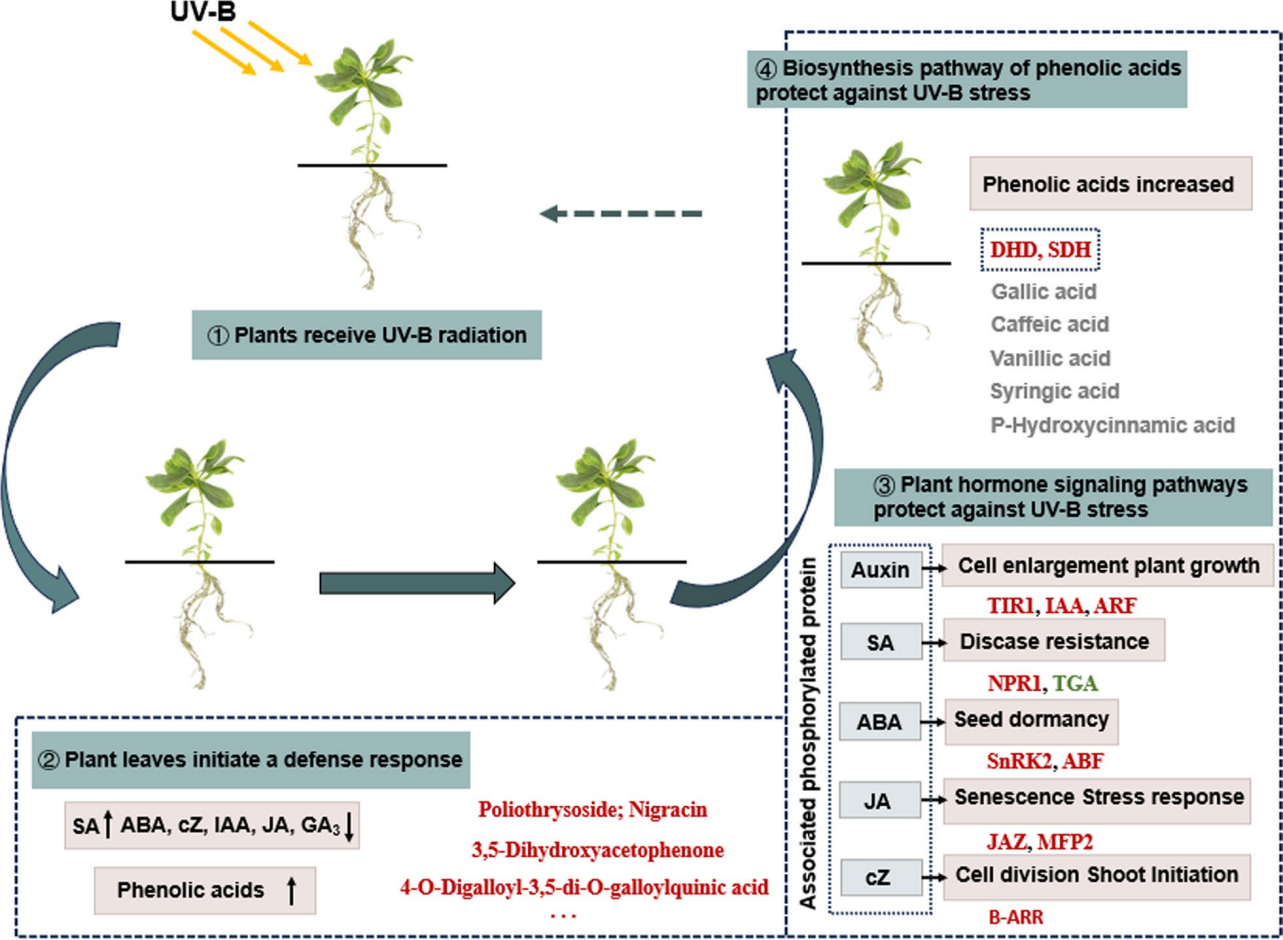
Mechanism of phenolic acid biosynthesis and plant hormone signal transduction pathway of *R. chrysanthum* resisting UV-B radiation

This experiment was conducted to reveal the regulatory network of phytohormones and phenolic acids in *R. chrysanthum* under UV-B stress. The physiology and biochemistry of *R. chrysanthum* were determined by UV-B radiation, and the changes of plant endogenous hormones and the correlation among hormones were compared. The results showed that phenolic acids and endogenous hormones were significantly changed and growth hormone was positively correlated with cytokinin in *R. chrysanthum* after UV-B radiation. Then, using the combination of phosphorylated proteome and metabolic group study, we screened out the main DAMs and DEPs between UV-B radiation resistance and phenolic acid biosynthesis pathway, plant hormone synthesis and plant hormone signal transduction, in an attempt to reveal the regulatory mechanism of *R. chrysanthum* seedlings under UV-B stress (Fig. 5). It laid a foundation for further study on the molecular mechanism of the effects of plant hormones and phenolic acids on plant radiation resistance to UV-B.

## Data Availability

The data used in this study are available from the corresponding author on submission of a reasonable request.
